# Ventilator management and risk of air leak syndrome in patients with SARS-CoV-2 pneumonia: a single-center, retrospective, observational study

**DOI:** 10.1186/s12890-023-02549-7

**Published:** 2023-07-10

**Authors:** Nodoka Miyake, Yutaka Igarashi, Ryuta Nakae, Taiki Mizobuchi, Tomohiko Masuno, Shoji Yokobori

**Affiliations:** Department of Emergency and Critical Care Medicine, Nippon Medical School Hospital, 1-1-5 Sendagi, Bunkyo-Ku, Tokyo, 113-8603 Japan

**Keywords:** Air leak syndrome, Coronavirus disease 2019, Ventilator management, Retrospective, Pneumonia

## Abstract

**Background:**

Severe acute respiratory syndrome coronavirus 2 (SARS-CoV-2) pneumonia is reportedly associated with air leak syndrome (ALS), including mediastinal emphysema and pneumothorax, and has a high mortality rate. In this study, we compared values obtained every minute from ventilators to clarify the relationship between ventilator management and risk of developing ALS.

**Methods:**

This single-center, retrospective, observational study was conducted at a tertiary care hospital in Tokyo, Japan, over a 21-month period. Information on patient background, ventilator data, and outcomes was collected from adult patients with SARS-CoV-2 pneumonia on ventilator management. Patients who developed ALS within 30 days of ventilator management initiation (ALS group) were compared with those who did not (non-ALS group).

**Results:**

Of the 105 patients, 14 (13%) developed ALS. The median positive-end expiratory pressure (PEEP) difference was 0.20 cmH_2_O (95% confidence interval [CI], 0.20–0.20) and it was higher in the ALS group than in the non-ALS group (9.6 [7.8–20.2] vs. 9.3 [7.3–10.2], respectively). For peak pressure, the median difference was -0.30 cmH_2_O (95% CI, -0.30 – -0.20) (20.4 [17.0–24.4] in the ALS group vs. 20.9 [16.7–24.6] in the non-ALS group). The mean pressure difference of 0.0 cmH_2_O (95% CI, 0.0–0.0) (12.7 [10.9–14.6] vs. 13.0 [10.3–15.0], respectively) was also higher in the non-ALS group than in the ALS group. The difference in single ventilation volume per ideal body weight was 0.71 mL/kg (95% CI, 0.70–0.72) (8.17 [6.79–9.54] vs. 7.43 [6.03–8.81], respectively), and the difference in dynamic lung compliance was 8.27 mL/cmH_2_O (95% CI, 12.76–21.95) (43.8 [28.2–68.8] vs. 35.7 [26.5–41.5], respectively); both were higher in the ALS group than in the non-ALS group.

**Conclusions:**

There was no association between higher ventilator pressures and the development of ALS. The ALS group had higher dynamic lung compliance and tidal volumes than the non-ALS group, which may indicate a pulmonary contribution to ALS. Ventilator management that limits tidal volume may prevent ALS development.

**Supplementary Information:**

The online version contains supplementary material available at 10.1186/s12890-023-02549-7.

## Background

Coronavirus disease 2019 (COVID-19), which originated in Wuhan, China, in 2019, rapidly spread worldwide. On March 11, 2020, the World Health Organization declared COVID-19 a pandemic, and as of August 2022, approximately 600 million people worldwide have been infected, causing a medical emergency. About 80% of patients are asymptomatic or have mild symptoms; however, some develop moderate or serious severe acute respiratory syndrome coronavirus 2 (SARS-CoV-2) pneumonia requiring invasive mechanical ventilation [1]. The overall fatality rate from COVID-19 is 2–3%; however, it rises to 30–60% if ventilatory management is required [2].

Patients with SARS-CoV-2 pneumonia are prone to the development of complications such as air leak syndrome (ALS), including mediastinal emphysema and pneumothorax [3]. The incidence of ALS in patients with COVID-19 on ventilators has been reported to be 13–19% [4, 5]. Because the mortality rate is higher in ventilated patients with SARS-CoV-2 pneumonia complicated with ALS than in uncomplicated cases [6], the goal is to use ventilators to reduce the incidence of ALS. ALS is generally caused by ventilator-induced lung injury (VILI) and alveolar changes [6]. Therapeutic strategies such as limiting tidal volume, plateau pressure, and driving pressure can help reduce the incidence of VILI [7, 8].

However, the impact of ventilator management in patients with SARS-CoV-2 pneumonia on ALS development is not well studied. Previous studies have only used data from initial ventilator settings and from 24 h prior to the onset of ALS and have failed to reflect the effects of changes in settings, cumulative pressure, and the pressure applied to the patient’s lungs by spontaneous breathing [9, 10]. To determine the impact of ventilator management on the development of ALS, this study utilized values obtained from ventilators every minute to examine factors associated with ALS development.

## Methods

### Study design

This was a single-center, retrospective, observational study conducted at a tertiary care hospital. Informed consent was obtained from patients via an opt-out method through the hospital website. The study was approved by the Nippon Medical School Hospital Ethics Committee (Study number: B-2022–555, Approval date: 2022.10.22, Study title: Study of complications in patients with COVID-19 using data from the critical care management system; Name of the governing board granting approval or waiver: Dr. Hiroki Yamaguchi), and the procedures followed were in accordance with the ethical standards of the responsible committee on human experimentation (institutional or regional) and with the most recent amendment of the Declaration of Helsinki, 1975.

### Settings

All adult patients who tested positive for SARS-CoV-2 with real-time reverse transcriptase-polymerase chain reaction assay, were diagnosed with pneumonia, and admitted to the intensive care unit (ICU) and ventilated between March 1, 2020, and November 30, 2021, were included. Ventilator management was started for patients in whom SpO_2_ was < 93% or PaO_2_ was < 60 mmHg even after the administration of 10 L of oxygen through a reservoir mask in accordance with the clinical practice guideline [11]. Comparisons were made between patients who developed ALS and those who did not. Patients with the following conditions were excluded: cardiopulmonary arrest at presentation, ALS at presentation, and missing data such as patient demographics and ventilator measurements.

### Data collection

The following data were extracted from the electronic medical records: patient demographics, treatments, laboratory exams and arterial blood gas findings on admission, ventilator measurements (positive end-expiratory pressure [PEEP], mean airway pressure, peak pressure, respiratory rate, tidal volume, and ventilation volume per minute), and outcome (ALS, discharged alive, ventilator-free days, and ICU stays). Vital signs and actual ventilator values were saved as minute-by-minute values displayed on the monitor. Dynamic compliance was calculated as ⊿V/⊿P = tidal volume/plateau pressure-PEEP. The outcome was the onset of ALS within ventilator liberation or 30 days after the start of ventilator management. Simple chest radiography was performed daily, and two certificated emergency physicians checked the images. A chest computed tomography (CT) was performed if ALS was suspected from the chest radiogram. ALS was diagnosed if there were findings of subcutaneous emphysema, mediastinal emphysema, or pneumothorax on a chest CT. If ALS occurred, data up to the day before ALS occurred were used. Missing values for ventilator measurements and vital signs were not complemented, and patients were excluded if more than 10% of the total data were missing.

### Statistical analysis

The values obtained from each patient’s ventilator were tabulated on a minute-by-minute basis and combined into a table for each group. The median values of pressure and volume on the ventilator and the number of times these values exceeded the reference values were calculated (Additional file 1). From the Acute Respiratory Distress Syndrome (ARDS) guidelines, peak pressure ≥ 40 cmH_2_O and tidal volume ≥ 8 mL per ideal body weight are harmful and deviate from recommendations. We counted the number of times that the tidal volume was harmful and compared this to the proportion of harmful tidal volumes during ventilator management.

Continuous variables are expressed as median and interquartile range. Categorical variables were analyzed using the χ-square test. For continuous variables, we used the t test for normally distributed data and the Mann–Whitney U test for non-normally distributed data. Statistical significance was defined as a *p*-value < 0.05. Statistical analysis was performed using R version 4.0.4 (The R Foundation for Statistical Computing, Vienna, Austria).

## Results

**Fig. 1:**
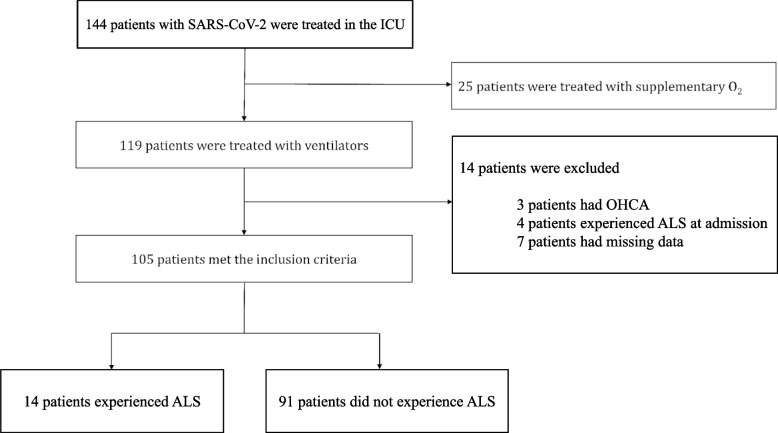
Inclusion criteria. ALS, air leak syndrome; COVID-19, coronavirus disease 2019; ICU, intensive care unit; OHCA, out-of-hospital cardiac arrest

During the study period, 144 patients with SARS-CoV-2 pneumonia were admitted to the ICU. Of the admitted patients, 119 received ventilator management. Excluding three patients with cardiopulmonary arrest on arrival, four patients who had ALS on arrival, and seven patients with missing data, 105 patients were included in the study (Fig. 1). Fourteen patients (13%) developed ALS within 30 days of starting ventilator management, and the median onset date was 14 [12-18] days after starting ventilator management.

### Patient characteristics

Baseline characteristics were not significantly different between the ALS and non-ALS groups, except for a higher rate of high-volume steroid use in the ALS group (57% vs. 22%, *p* = 0.01) (Table 1).

**Table 1:** Characteristics, medical history, treatment, and laboratory findings

Variables	ALS (*n* = 14)	Non-ALS (*n* = 91)	*P* value
Age (years), median ± IQR	66.4 ± 9.9	61.4 ± 15.5	0.24
Sex (male), *n* (%)	12 (86)	66 (73)	0.47
BMI, median ± IQR	25.0 ± 4.1	26.6 ± 5.4	0.30
Past history			
Hypertension,* n* (%)	6 (43)	45 (50)	0.86
Diabetes, *n* (%)	2 (14)	35 (39)	0.14
Heart failure, *n* (%)	2 (14)	10 (11)	1.00
Chronic obstructive pulmonary disease, *n* (%)	1 (7)	2 (2)	0.35
Interstitial pneumonia, *n* (%)	0 (0)	2 (2)	1.00
Kidney disease, *n* (%)	1 (7)	5 (6)	1.00
Hematologic disease, *n* (%)	0 (0)	2 (2)	1.00
Psychiatric disease, *n* (%)	0 (0)	7 (8)	0.62
PaO_2_/FiO_2_ ratio, median (95% CI)	68.0 (57.3–118.4)	84.3 (63.9–122.5)	0.92
Laboratory			
WBC (/µL), median (95% CI)	9,750 (8,200–11,700)	7,950 (6,280–10,780)	0.22
Hb (g/dL), median (95% CI)	14.4 (12.2–15.5)	13.2 (11.6–14.3)	0.26
Plt (10^4^/µL), median (95% CI)	19.8 (16.4–24.7)	20.6 (16–26.6)	0.99
D-dimer (µg/mL), median (95% CI)	1.8 (1.5–3.3)	1.5 (1.2–2.7)	0.15
CRP (mg/dL), median (95% CI)	8.8 (5.2–12.0)	10.2 (4.4–16.0)	0.63
LDH (IU/L), median (95% CI)	487.5 (399.0–639.5)	462.0 (385.3–611.5)	0.70
AST (IU/L), median (95% CI)	61.0 (42.0–74.3)	46.0 (34.8–66.0)	0.33
ALT (IU/L), median (95% CI)	45.5 (34.8–104.5)	41.0 (23.8–60.5)	0.18
Treatment			
Prone position, *n* (%)	8 (57)	30 (33)	0.15
ECMO, *n* (%)	2 (14)	6 (7)	0.64
CHDF, *n* (%)	5 (36)	19 (21)	0.37
Dexamethasone, *n* (%)	14 (100)	76 (84)	0.22
Steroid pulse, *n* (%)	8 (57)	20 (22)	0.01
Remdesivir, *n* (%)	10 (71)	74 (81)	0.62
Baricitinib, *n* (%)	2 (14)	27 (30)	0.38
Catecholamine, *n* (%)	10 (71)	45 (50)	0.21
Rocuronium, *n* (%)	3 (21)	9 (10)	0.42
Outcomes			
ICU days, *n* (%)	40.00 (14.89)	17.07 (13.01)	< 0.001
Mechanical ventilator days, *n* (%)	37.57 (14.61)	14.12 (13.76)	< 0.001
Dead, *n* (%)	6 (43)	11 (12)	0.01

*ALS* Air leak syndrome, *ALT* Alanine transaminase, *AST* Aspartate transaminase, *BMI* Body Mass Index, *CHDF* Continuous hemodiafiltration, *CI* Confidence interval, *CRP* C-reactive protein, *ECMO* Extracorporeal Membrane Oxygenation, *Hb* Hemoglobin, *ICU* Intensive care unit, *IQR* Interquartile range, *LDH* Lactate dehydrogenase, *Plt* Platelet, *WBC* White blood count

### Analysis of ventilators

The total ventilator time in the ALS group and the non-ALS group was 172.2 days (247,987 min) and 980.4 days (1,411,736 min), respectively, with a mean per patient of 12.3 ± 6.2 vs. 11.1 ± 6.9 days (*p* = 0.30), respectively. Peak pressure and mean airway pressure were 0.30 cmH_2_O (95% confidence interval [CI], 0.30–0.20) and 0.20 cmH_2_O (95% CI, 0.20–0.20), respectively and these were higher in the non-ALS group. Tidal volume (59.0 mL [95% CI, 58.0–60.0]), single ventilation volume per ideal body weight (0.71 mL/kg [95% CI, 0.70–0.72]), minute volume (0.80 L/min [95% CI, 0.80–0.80]), and arterial lung compliance were higher in the ALS group (8.27 [95% CI, 12.76–21.95]) than in the non-ALS group (Table 2).

**Table 2:** Ventilator data per minute for patients with/without air leak syndrome

Variables	ALS (*n* = 247,987)	Non-ALS (*n* = 1,411,736)	Differences (95% CI)	P value
Median peak (cmH_2_O), median (95% CI)	20.4 (17.0–24.4)	20.9 (16.7–24.6)	-0.30 (-0.30 – -0.20)	< 0.001
Median PEEP (cmH_2_O), median (95% CI)	9.6 (7.8–10.2)	9.3 (7.3–10.2)	0.20 (0.20–0.20)	< 0.001
Median MP (cmH_2_O), median (95% CI)	12.7 (10.9–14.6)	13.0 (10.3–15.0)	0 (0–0)	< 0.001
Median respiratory rate (/min), median (95% CI)	17.4 (14.9–21.5)	18.5 (15.9–22.0)	-1.0 (-1.0 – -1.0)	< 0.001
Median tidal volume (mL), median (95% CI)	501.0 (411.0–600.0)	442.0 (359.0–529.0)	59.0 (58.0–60.0)	< 0.001
Median TV/IBW (mL/kg), median (95% CI)	8.17 (6.79–9.54)	7.43 (6.03–8.82)	0.71 (0.70–0.72)	< 0.001
Median Cdyn (mL/cmH_2_O), median (95% CI)	43.8 (28.2–68.8)	35.7 (26.5–41.5)	8.27 (12.76–21.95)	< 0.001
Median MV (L/min), median (95% CI)	8.60 (7.20–10.60)	7.96 (6.60–9.40)	0.80 (0.80–0.80)	< 0.001
Number of peaks ≥ 40 cmH_2_O, *n* (%)	24 (0.0)	5,859 (0.4)	NA	< 0.001
Number of TV/IBW ≥ 8 mL/kg, *n* (%)	132,949 (53.7)	537,674 (38.6)	NA	< 0.001

*ALS* Air leak syndrome, *Cdyn* Dynamic compliance, *CI* Confidence interval, *MP* Mean pressure, *PEEP* Positive end-expiratory pressure, *TV/IBW* Tidal volume/ideal body weight, *MV* Minute volume

The frequency of a peak pressure > 40 mmHg was higher in the non-ALS group than in the ALS group (0.0% vs. 0.4%, respectively, *p* < 0.001), but the frequency that the ventilation volume per body weight exceeded eight was greater in the ALS group than in the non-ALS group (53.7% vs. 38.6%, respectively, *p* < 0.001).

In the ALS group, the mean peak pressure was 1.58 (95% CI, 1.47–1.67) cmH_2_O, the mean PEEP was 0.06 (0.03–0.10) cmH_2_O, and the mean airway pressure was 0.51 (0.45–0.56) cmH_2_O, which were higher during the 24 h prior to ALS onset (Additional file 2).

### Outcomes

The ALS group had a longer duration of ventilator use (38 ± 15 days vs. 14 ± 14 days, *p* < 0.001), longer ICU stays (40 ± 15 days vs. 17 ± 14 days, *p* < 0.001), and higher mortality (43% vs. 12%, *p* = 0.01) than the non-ALS group (Table 1).

## Discussion

In this study, we analyzed minute-by-minute ventilator data from patients with SARS-CoV-2 pneumonia over a total of more than 1000 days and analyzed the association with ALS development. Although there was no association between high ventilator pressure and ALS, there was an association between high tidal volume and high dynamic lung compliance and ALS development. In addition, mortality was higher for those who developed ALS.

ALS is a common high-risk complication of ARDS; 13–15% of patients with ARDS on ventilator management develop ALS [12, 13]. High tidal volume and high driving pressure are known risk factors for ALS [14]. A tidal volume of around 6 mL/kg is recommended so that high driving pressure does not increase tidal volume [8, 15]. However, in a previous study on patients with SARS-CoV-2 pneumonia, the initial settings of PEEP and tidal volume were not associated with the development of ALS [9]; however, the effect of changes in settings, cumulative pressure, and actual pressure due to spontaneous breathing was not considered. The results of the present study, using minute-by-minute measurements, revealed that the peak and mean airway pressures were rather lower in the ALS group, and PEEP was only 0.20 cmH_2_O higher; this is a small difference because the pressures of most ventilators are set in units of 1 cmH_2_O.

SARS-CoV-2 pneumonia is clinically different than ARDS. In ARDS, pulmonary compliance is reduced by decreased ventilatory lung volume due to effusions in the interstitial and alveoli and decreased pulmonary surfactant. Contrarily, early-stage SARS-CoV-2 pneumonia is characterized by severe hypoxemia (silent or happy hypoxia) without dyspnea [9, 16], with no increase in the breathing workload because lung compliance is not significantly reduced [9, 16]. However, patients with ARDS complicated by COVID-19 have a higher incidence of ALS compared to patients with ARDS due to causes other than COVID-19. ALS is reported to occur seven times more frequently in patients with ARDS due to causes other than COVID-19 [5].

Pathologically, COVID-19 reportedly causes diffuse alveolar damage, hyaline membrane formation, lymphocyte and/or monocyte infiltration, and pneumocyte hyperplasia [17]. However, these findings do not explain the clinical, radiological, and physiological features observed in SARS-CoV-2 pneumonia, findings that are also observed in patients with ARDS. Autopsies of patients with COVID-19 are likely to show signs of ARDS, a complication with high mortality rates. Therefore, it is difficult to explain the difference in pulmonary compliance between COVID-19 and ARDS by pathology.

Both COVID-19 and ARDS are associated with ALS development; however, ALS development in COVID-19 suggests that factors on the pulmonary side of the patient are more likely to cause ALS than the ventilator pressure settings. Randomized controlled trials are needed to clarify whether tidal volume-limited ventilator management can prevent ALS.

### Limitations

First, due to the retrospective nature of this study, we could not determine what caused ALS development. Although the use of steroid pulses was significantly different in the ALS and non-ALS groups at baseline and may have influenced ALS development, the severity of pneumonia poses a potential bias in terms of the administration of steroid pulses. There have been reports of mediastinal emphysema complications during steroid treatment for collagen diseases [18, 19], and the possibility that delayed tissue repair caused by steroid drugs may cause ALS has been considered. However, another study did not reveal any statistical association between steroid pulse and ALS for patients with ARDS by multiple logistic regression analysis [20]. Second, there were 10 patients in the non-ALS group who were transferred to other hospitals on a ventilator within 30 days and it was not known whether these patients developed ALS. These patients were considered at low risk of developing ALS after 15 days due to their improved condition and minimal ventilatory support. Third, values per minute were used; however, events of < 1 min (e.g., bucking or suction by nurses) were not recorded in the data, even if they affected ALS development. Fourth, minor ALS events that cannot be diagnosed by chest X-ray may have been missed; however, these are not clinically important. Finally, the study did not consider differences in the pathogenesis of different virus strains.

## Conclusions

We analyzed the association between ventilator management and ALS development in patients with SARS-CoV-2 pneumonia using minute-by-minute values obtained from ventilators. Randomized controlled trials are needed to clarify whether ventilator management with limited tidal volume can prevent the development of ALS.

## Supplementary Information

**Additional file 1.** Methods for collecting and analyzing ventilator data on a minute-by-minute basis.

**Additional file 2.** Ventilator data per minute for patients with /without air leak syndrome of 24 hours prior to the onset of ALS.

## Data Availability

The datasets generated and analyzed during the current study are available from the corresponding author on reasonable request.

## Abbreviations

ALS: Air leak syndrome
ARDS: Acute respiratory distress syndrome
COVID-19: Coronavirus disease 2019
CT: Computed tomography
ICU: Intensive care unit
PEEP: Positive-end expiratory pressure
SARS-CoV-2: Severe acute respiratory syndrome coronavirus 2
VILI: Ventilator-induced lung injury
