# Supplementary material for: Ventilator management and risk of air leak syndrome in patients with SARS-CoV-2 pneumonia: a single-center, retrospective, observational study
Source: BMC Pulm Med. 2023 Jul 10;23:251. doi: 10.1186/s12890-023-02549-7 (PMC10334538; doi:10.1186/s12890-023-02549-7)
Supplement: Supplementary file 1 — Additional file 1. Methods for collecting and analyzing ventilator data on a minute-by-minute basis. [file 12890_2023_2549_MOESM1_ESM.pdf]

Additional file 1

Methods for collecting and analyzing ventilator data on a minute-by-minute basis

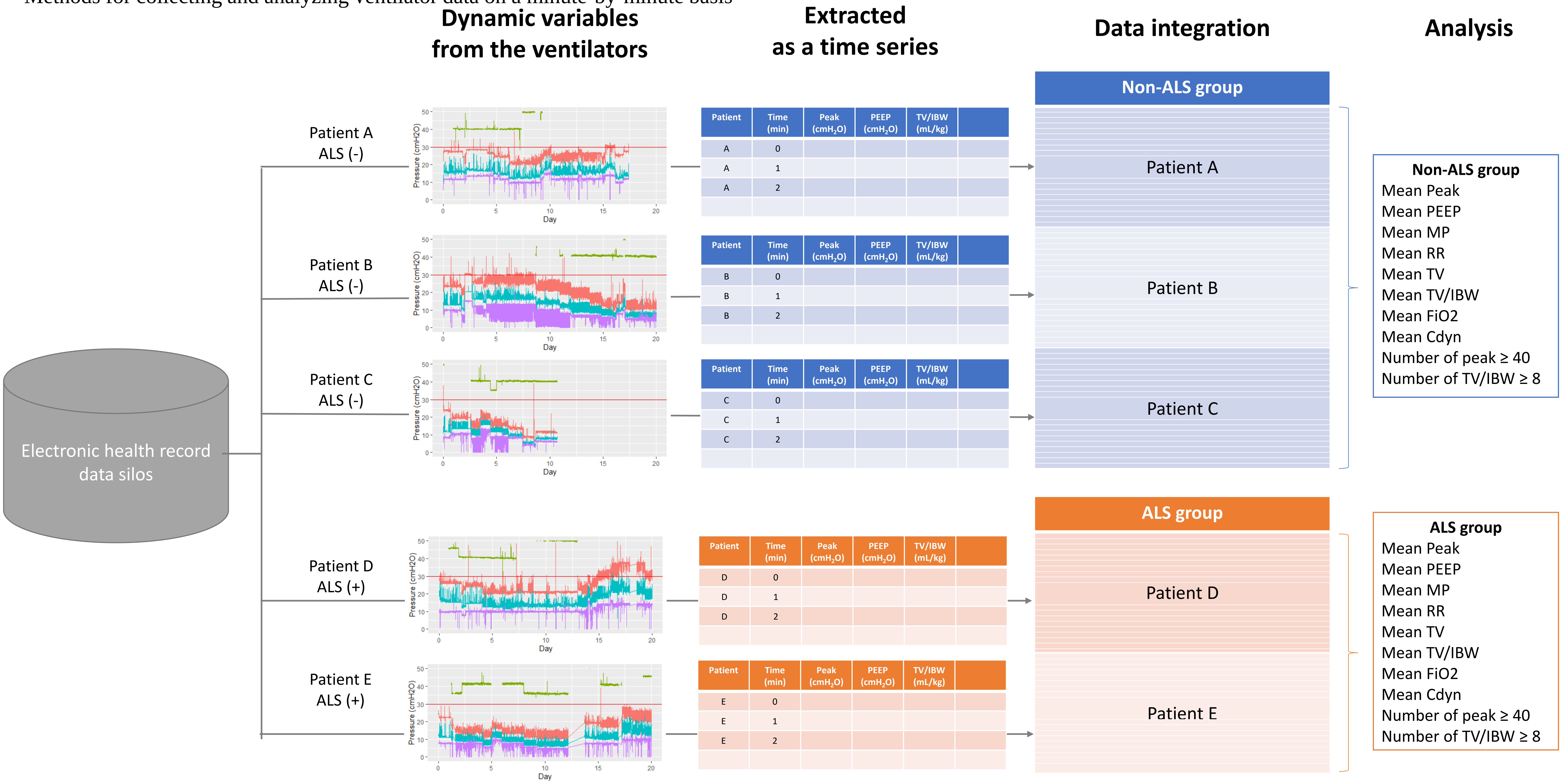

The values obtained from each patient's ventilator were tabulated on a minute-by-minute basis and combined into a table for each group. The mean values of pressure and volume on the ventilator and the number of times these values exceeded the reference values were calculated.
