# Supplementary material for: Ventilator management and risk of air leak syndrome in patients with SARS-CoV-2 pneumonia: a single-center, retrospective, observational study
Source: BMC Pulm Med. 2023 Jul 10;23:251. doi: 10.1186/s12890-023-02549-7 (PMC10334538; doi:10.1186/s12890-023-02549-7)
Supplement: Supplementary file 2 — Additional file 2. Ventilator data per minute for patients with /without air leak syndrome of 24 hours prior to the onset of ALS. [file 12890_2023_2549_MOESM2_ESM.pdf]

Additional file 2

Ventilator data per minute for patients with /without air leak syndrome of 24 hours prior to the onset of ALS

Variables	Within 24 h before ALS (*n*=19,644)	Other period (*n*=249,427)	Differences (95% confidence interval)	P value
Mean peak (cmH_2O)	22.27 ± 6.75	20.69 ± 5.29	1.58 (1.47–1.67)	< 0.001
Mean PEEP (cmH_2O)	9.24 ± 2.49	9.18 ± 2.20	0.06 (0.03–0.10)	< 0.001
Mean MP (cmH_2O)	13.30 ± 3.99	12.79 ± 3.12	0.51 (0.45–0.56)	< 0.001
Mean respiratory rate (/min)	22.58 ± 9.64	18.45 ± 6.03	4.13 (4.00–4.27)	< 0.001
Mean tidal volume (mL)	446.00 ± 194.23	488.21 ± 205.66	-0.55 (39.38–45.04)	< 0.001
Mean TV/IBW (mL/kg)	7.27 ± 2.89	7.82 ± 3.16	-8.23 (0.60–0.51)	< 0.001
Mean Cdyn (mL/cmH_2O)	42.00 ±28.16	50.23 ± 31.62	-8.23 (7.82–8.65)	< 0.001
Mean MV (L/min)	9.59 ± 3.89	8.89 ± 2.75	0.70 (0.65–0.76)	< 0.001
Number of peak ≥ 40 cmH_2O, *n* (%)	5 (0.0)	24 (0.0)	NA	< 0.001
Number of TV/IBW ≥ 8 mL/kg, *n* (%)	5,611 (28.6)	133,261 (53.5)	NA	< 0.001

In the ALS group, the mean peak pressure was 1.58 (95% CI, 1.47–1.67) cmH_2O, the mean PEEP was 0.06 (0.03–0.10) cmH_2O, and the mean airway pressure was 0.51 (0.45–0.56) cmH_2O, which were higher in the 24 hours prior to the onset of ALS.
